# The Ginsenoside Rg_1_ Rescues Mitochondrial Disorders in Aristolochic Acid-Induced Nephropathic Mice

**DOI:** 10.3390/life11101018

**Published:** 2021-09-27

**Authors:** Chu-Kuang Chou, Yu-Shen Huang, Pei-Yu Lin, Kazuhiro Imai, Shih-Ming Chen, Jen-Ai Lee

**Affiliations:** 1 Division of Gastroenterology and Hepatology, Ditmanson Medical Foundation Chia-Yi Christian Hospital, Chiayi 60002, Taiwan; vacinu@gmail.com; 2 School of Pharmacy, College of Pharmacy, Taipei Medical University, Taipei 11031, Taiwan; danny-ccl.huang@sgs.com (Y.-S.H.); smchen@tmu.edu.tw (S.-M.C.); 3 Department of Pharmacy, National Yang Ming Chiao Tung University Hospital, Yilan 26042, Taiwan; 15132@ymuh.ym.edu.tw; 4 Research Institute of Pharmaceutical Sciences, Musashino University, Tokyo 180-8777, Japan; k-imai@musashino-u.ac.jp

**Keywords:** aristolochic acid, nephropathy, ginsenoside, Rg_1_, mitochondrial disorder

## Abstract

Chronic exposure to aristolochic acid (AA) leads to renal interstitial fibrosis and nephropathy. In this study, we aimed to investigate the renoprotective effects of *Panax ginseng* extract (GE) and ginsenoside saponin (GS) on AA-induced nephropathy (AAN) in mice. Eighty female C3H/He mice were randomly divided into eight groups, including normal; AA (3 μg/mL for 56 days); AA with GE (125, 250, or 500 mg/kg/d for 14 days); and AA with important GE ingredients, Rg_1_, Rb_1_, or Rd (5 mg/kg/d for 14 days). Compared with the AA group, renal injuries were significantly decreased in the GE (250 mg/kg/d), Rb_1_, and Rg_1_ treatment groups. Rg_1_ exhibited the best renoprotection among all GS-treated groups. There were 24 peaks significantly altered among normal, AA, and AA + Rg_1_ groups, and four mitochondrial proteins were identified, including acyl-CoA synthetase medium-chain family member 2, upregulated during skeletal muscle growth 5 (Usmg5), mitochondrial aconitase 2 (ACO2), and cytochrome c oxidase subunit Va preprotein (COX5a). We demonstrated for the first time that the AAN mechanism and renoprotective effects of Rg_1_ are associated with expression of mitochondrial proteins, especially ACO2, Usmg5, and COX5a.

## 1. Introduction

In the 20th century, rapidly progressive tubulointerstitial nephritis was reported in female patients who followed slimming regimens containing aristolochic acid (AA). According to National Health Insurance (NIH) data, about one-third of Taiwanese people were prescribed with AA-containing products between 1997 and 2003 [1]. Although AA-containing products are prohibited in various countries, AA-induced nephropathy (AAN) is still frequently reported all over the world, especially in Asian populations [2]. Aristolochic acid I (AAI) and aristolochic acid II (AAII) are the major components of the AA mixture in the plant extracts. However, AAI is more toxic than AAII due to the substitution of the methoxyl group. Intraperitoneal injection of AA induces acute kidney injury (AKI), which leads to progressive chronic kidney disease (CKD) in mice [3]. In AA-induced AKI mice, oxidative stress was associated with inflammation [4]. The proximal renal tubules are the primary cellular target of AA, especially in the proximal tubular S3 segment [5,6].

Recent studies have confirmed that AA can induce severe mitochondrial damage and condense mitochondrial membrane density through increasing the levels of superoxide anion, ferrous ions, and reactive oxygen species (ROS); lowering mitochondrial membrane potential (MMP); decreasing mitochondrial DNA (mtDNA) copy number; reducing adenosine triphosphate (ATP) production; and activating the renal mitochondrial apoptosis pathway [7,8]. The mechanisms of AAN include elevation of oxidative stress, advanced glycation end-products, apoptosis, inflammation, and renal interstitial fibrosis [2,9,10]. More recently, activating the mammalian target of rapamycin (mTOR)-autophagy axis has been demonstrated to remove AA-damaged mitochondria and misfolded proteins, which can attenuate AAN in mice [11]. Therefore, mitochondria-related proteins might have a crucial role in AAN mice. However, the detailed mechanism of how AA induces renal injury still needs to be investigated, and there is still no effective treatment for AAN.

*Panax ginseng* C.A. Meyer is one of the species that has been commonly used as a tonic in Eastern Asia for thousands of years [12]. In Asia, two types of commercial ginseng products are available in herbal medicine, including white and red ginseng. The steaming and drying of red ginseng is more useful in terms of the resulting pharmacological profile than the same for white ginseng [13]. The protective effects of the ginseng extract (GE) include anti-apoptosis, antioxidant activity, renoprotection, and anti-inflammation in vitro and/or in vivo [14,15,16,17]. However, ginsenosides (GS), the bioactive compounds in *Panax ginseng*, have received more attention recently. According to its aglycone moieties, GS can be classified into two main categories: Dammarane-type triterpene saponins with 20 (*S*)-protopanaxadiol (Rb_1_, Rb_2_, Rc, Rd, Rg_3_, and Rs_3_); 20 *(S)*-protopanaxatriol (Re, Rg_1_, and Rf) as the aglycone. Among them, Rg_1_, Rd, and Rb_1_ are three of the most important ingredients in *Panax ginseng* [18]. Notably, Rg_1_, and Rb_1_ inhibit renal interstitial fibrosis in rats with unilateral ureteral obstruction [19,20]. Rg_1_ could protect podocytes from sMAC-induced injury [21]. Ginsenoside Rg_3_ attenuated cisplatin-induced apoptosis and damage, decreased the proportion of late apoptotic cells, elevated mitochondrial membrane potential, and ameliorated histopathological damage in the kidney [22]. Rd modulates the macrophage phenotype, which alleviates acute renal ischemia/reperfusion injury [23]. Additionally, red ginseng significantly ameliorated gentamicin (GM)-induced ROS production, which was associated with the protection of renal tubular cells from apoptosis that resulted in significant amelioration of GM-induced AKI [24].

The present study investigated the underlying molecular mechanisms of action of GE and its bioactive ingredients, Rg_1_, Rd, and Rb_1_, against AAN. Renal differential proteomics was applied to discover the altered proteins in AAN mice with Rg_1_ treatment. The findings suggested that GE and its bioactive ingredients, Rg_1_, Rd, and Rb_1_, attenuated AAN by expression of mitochondrial proteins.

## 2. Materials and Methods

The AA sodium salt (AAI 63%, AAII 31%), 10% buffered neutral formalin, calcium chloride, tris(2-carboxyethyl)phosphine (TCEP), ammonium bicarbonate, and guanidine buffer guanidine hydrochloride 8 M were purchased from Sigma (St. Louis, MO, USA). *Ginseng Radix Rubra* was purchased from the Chinese herbal medicine store (Chang Sheng, Taipei, Taiwan). The ginsenoside Rg_1_ was purchased from Nacalai Tesque (Kyoto, Japan). Ginsenosides Rb_1_ and Rd were purchased from Extrasynthèse (Genay, France). HPLC-grade acetonitrile (ACN) was purchased from Merk (Darmstadt, Germany). High performance liquid chromatography (HPLC)-grade isopropanol (IPA) was purchased from Mallinckrodt Baker Inc. (Lexington, KY, USA). Ethylenediaminetetraacetic acid disodium salt (EDTA 2Na) and 3-[(3-cholamidopropyl)dimethylammonio]propansesulfonic acid (CHAPS) were purchased Wako Pure Chemical (Osaka, Japan). Trifluoroacetic acid (TFA) was purchased from Alfa Aesar (Heysham, UK). Sequencing grade trypsin was purchased from Promega (Fitchburg, WI, USA). 4-[2-(Dimethylamino)-ethylaminosulfonyl]-7–chloro -2,1,3-benzoxadiazole (DAABD-Cl) was purchased from Tokyo Chemical Industry Inc. (Tokyo, Japan).

### 2.1. Preparation of the Ginseng Extract

The extraction of *Panax ginseng* was performed following the protocol published by Kim et al. with a minor modification [25]. Six-year-old *Ginseng Radix Rubra* (300 g) was boiled for 6 h in 50% ethanol (1:10, *w*/*v*), and we collected the filtrate in triplicate. The filtrate was concentrated using rotary evaporation and was lyophilized. The overall yield of GE was about 39% of the dry weight of *Ginseng Radix Rubra*.

### 2.2. Animal Experiments and Sample Collection

Six-week-old female C3H/e mice were purchased from the National Laboratory Animal Center (Taipei, Taiwan) and maintained in the Laboratory Animal Center of Taipei Medical University. All animal experiments were reviewed and approved by the Institutional Animal Care and Use Committee or Panel (permit number: LAC-101-0318) to minimize pain and discomfort. Mice were given feed and water ad libitum. The mice were randomly divided into eight groups (*n* = 10/each), including normal; AA; AA with GE (125, 250 m or 500 mg/kg/d); and Rg_1_, Rb_1_, or Rd 5 mg/kg/d treatment. In the AA group, AA sodium salt was dissolved in distilled drinking water (3 μg/mL, 0.5 mg/kg/d) for 56 days [26]. After 56 days, drinking water was replaced by distilled water for another 14 days. In treatment groups, after AA induction for 56 days, 0.1 mL of GE or GS was given orally for the next 14 days. The normal group was given distilled water during the experiment.

Twelve-hour urine was collected at day 70. At the end of the experiment, mice were sacrificed. Blood and kidney tissues were collected. Blood was collected from the tail vein and left at 25 °C for clotting. Afterwards, blood was centrifuged at 4 °C, 1006× *g* for 15 min, and then the supernatant was collected as the sera. The kidneys of mice were removed, rinsed with 0.9% normal saline, decapsulated, and fixed in 10% buffered neutral formalin, followed by dehydration in a gradient ethanol solution, and xylene clearing. Subsequently, kidneys were embedded in paraffin.

### 2.3. Histological Examination

Kidney sections (4–5 μm) were used to observe the renal injury with periodic acid and Schiff’s stain (Sigma, St. Louis, MO, USA), and the images were captured using OPTIMA G-330 light microscopy with a Nikon Coolpix 4500 camera (Digisystem, Taipei, Taiwan)(magnification: 200×). The tubulointerstitial histological scores of 20 images were averaged and evaluated for three parameters, including tubular atrophy (0–3), inflammatory cell infiltration (0–3), and interstitial fibrosis (0–3) [27,28].

### 2.4. Determination of Clinical Biochemistry

Serum creatinine level was determined using synchron creatinine reagent (Bechman Coulter, Carlsbad, CA, USA). Blood urea nitrogen (BUN) level was determined using the Beckman BUN kit (Sigma, St. Louis, MO, USA). Urinary N-acetyl-β-D-glucosaminidase (NAG) was measured using the fluorescent 4-methylumbelliferone (4-MU) (Sigma, St. Louis, MO, USA) [29,30]. The substrate (4-methylumbelliferyl *N*-acetyl-β-d-glucosaminide) was split via NAG to form 4-MU and 4-MU, reflecting the NAG activity, and the absorbance was measured at 370/460 nm using a plate reader (BMG Labtech Inc., Offenburg, Germany).

Urinary protein level was determined using Bio-Rad protein assay kits (Bio-Rad, Hercules, CA, USA) according to the Bradford method [31]. Bovine serum albumin (Bio-Rad Inc., Herecules, CA, USA) was used as a standard solution, and the protein content was positively correlated with the O.D. at 590 nm.

### 2.5. Determination of Rg_1_, Rb_1_, and Rd in GE

Rg_1_, Rb_1_, and Rd were quantified in GE through HPLC coupled with a UV–visible detector (Hitachi, Tokyo, Japan). The GE sample was dissolved in ddH_2_O (100 mg/mL, *w*/*v*) and filtrated through a Millipore 0.45 μm syringe filter (Millipore, Darmstadt, Germany). The resultant filtrate (30 μL) was directly injected to the HPLC system, followed by separation on an Intertsil ODS-2 column (4.6 × 150 mm I.D., 5 μm) (GL Sciences Inc., Tokyo, Japan) at 25 °C. The mobile phases of Rg_1_, and Rb_1_ and Rd were 20% or 30% aqueous ACN, respectively. The flow rate was set as 1 mL/min. The wavelength was 203 nm. All HPLC equipment was purchased from Hitachi (Tokyo, Japan). All HPLC methods were validated, in terms of the precision of intra-assay, inter-assay (CV < 15%), and accuracy (85–115%).

### 2.6. Tissue Homogenization and Derivatization of Renal Protein

Kidney tissues (50 mg) were homogenized in 500 μL of 10 mM aqueous CHAPS with Precellys Beads kits (Bertin Technologies, Tarnos, Germany) using Precellys^®^24 (Bertin Technologies, Tarnos, France). The homogenate was centrifuged at 8000 rpm for 30 s at 4 °C using a Mikro 22R Centrifuge (Model D-78532, Hettich zentrigugen, Tuttlingen, Germany). The supernatant was collected and stored at −80 °C for the following experiments.

The homogenate was diluted with 10 mM aqueous CHAPS to 4 mg/mL. Aliquots of diluted homogenate (10 μL) were mixed with 60 μL of 10 mM EDTA∙2Na, 50 mM CHAPS, 2.5 mM TCEP, 25 μL of 8 M guanidine-HCl buffer, and 5 μL of 140 mM DAABDCl in ACN, followed by heating at 40 °C for 10 min. TFA (20%, 3 μL) was added to stop the derivatization reaction.

### 2.7. Fluorogenic Derivatization-Liquid Chromatography-Tandem Mass Spectrometry (FD-LC/MS/MS)-Based Renal Proteomics

The FD-LC system, consisting of an L-2130 Intelligent Pump, an L-2200 Intelligent Autosampler, and an L-2485 Fluorescence Detector (FD, Hitachi, Tokyo, Japan), was used to separate the mixture protein in a WX-RP column (4.6 × 250 mm I.D., 3 μm) (Imtakt Co., Kyoto, Japan). Twenty microliters of the fluorogenic sample was injected into the HPLC column at 0.55 mL/min at 60 °C. The wavelength of FD-LC was set at 395 nm for excitation and 505 nm for emission. The mobile phases consisted of the following: (A) ACN:IPA:H_2_O:TFA (9:1:90:0.15); (B) ACN:IPA:H_2_O:TFA (69:1:30:0.15); (C) ACN:IPA:H_2_O:TFA (9:1:90:0.2). The gradient was set with the following elution: 5% B and 1% C held for 10 min; 30% B and 35% C for 5 min and held for 15 min; 35% B and C for 10 min; 38% B and 35% C for 20 min; 44% B and 55% C for 30 min and held for 50 min; 47% B and 53% C for 10 min; 48% B and 52% C for 45 min; 51% B and 49% C for 25 min; 60% B and 40% C for 200 min; 70% B and 30% C for 80 min; 90% B and 10% C for 30 min and held for 30 min; 100% B.

The significantly altered peaks from FD-LC were collected. The eluates were concentrated and dried using Savant Speed Van (Model SPD111V, Savant Instruments, Inc., Holbrook, NY, USA), and then digested with 2.5 μL of sequencing grade trypsin, 10 mM CaCl_2_, and 20 μL 50 mM NH_4_HCO_3_ at 37 °C for 2 h. The digested samples were dried and concentrated as before, and then refilled with 10 μL of mobile phase (A) for protein identification. The MS system that consisted of API 4000Q TRAP, Aglient 1100, and 1200 (Agilent, Santa Clara, Germany) was used to identify proteins. After biological samples were injected into the MS system, these were condensed in a guard column (5 × 0.3 mm I.D., particle size 5 μm) (ZORBAX 300SB-C18, Agilent Santa Clara, Germany) and then separated with a C_18_ column (75 μm × 150 mm I.D., particle size 5 μm) (CVC Micro-Tech Scientific Inc., Fontana, CA, USA). Subsequently, MASCOT searching engine with NCBInr database was applied for protein identification.

### 2.8. Isolation of Renal Mitochondria for Western Blot Analyses

Mitochondria were extracted using a previously published protocol [32]. The renal homogenates were centrifuged at 600× *g* for 10 min, and the supernatant (S1) was collected. S1 was centrifuged at 9000× *g* for 10 min, and the supernatant was removed. The pellet (P1) that contained mitochondria was resuspended in the mitochondrial buffer (200 μL of 250 mM sucrose, 10 mM Tris-HCl, pH 7.6, and 1 mM Na_2_EDTA) and centrifuged at 9000× *g* for 10 min. The pellet (P2) was resuspended in the mitochondrial buffer (200 μL). Protein concentrations were determined using the Pierce™ BCA protein assay kit (Thermo Scientific, Waltham,, IL, USA).

The renal mitochondrial proteins (9.6 μg) were separated using 10% SDS-PAGE and transferred to a 0.22 μm Immuno-Blot^TM^ polyvinylidene difluoride membrane (Bio-Rad Inc., Herecules, CA, USA) in the transfer buffer (25 mM Tris, 192 mM glycine, 20% MeOH) following a semi-dry transfer (55 mA, 40 min). Membranes were immersed in a blocking solution consisting of 10% skimmed milk in TBS-T (24.8 mM Tris, 150 mM NaCl, 2.7 mM KCl, 0.2% Tween-20, pH 7.4) for 1 h at 25 °C. Membranes were washed four times for 5 min in TBS-T. Membranes were incubated separately with rabbit polyclonal antibodies raised against the Aconitase 2 (ACO2) (ABclonal, dilution 1:1000) or β-actin (Proteintech, dilution 1:2000). Immunoblots were washed four times for 5 min in TBS-T and incubated for 1 h with an HRP-conjugated goat anti-rabbit IgG (Proteintech, diluted 1:4000) in TBS-T. Immunoblots were washed four times for 5 min in TBS-T, and specific proteins were observed using TOOLSensitive ECL (Biotools, Taipei, Taiwan) and imaged using GeneGnome5 imager ver.1.3.3.0 (Syngene, Frederick, MD, USA). The intensity of the band was measured using the GeneTool software ver. 4.02 (Syngene, MD, USA).

### 2.9. Multivariate and Univariate Statistical Analysis

Multivariate statistical analysis (Metaboanalyst 4.0) was used to evaluate the renal differential proteomics [33]. Partial least squares discriminant analysis (PLS-DA) was one of the supervised methods that were used to predict the significant observation. Due to PLS-DA having an overfitting problem, the validation of the PLS-DA model was necessary. In leave-one-out cross-validation (LOOCV), the difference between R^2^ and Q^2^ was less than 0.3, meaning the PLS-DA model was suitable for the discovery of the potential feature in omics. The normality of distribution was analysis with Metaboanalsyt online. The distribution normalization to sample median is shown in box plots in the Supplementary Material (Figure S1).

All experimental data are expressed as mean ± standard deviation. Two-tailed Mann–Whitney U tests were used to analyze the significant differences between pairs of groups. A *p*-value of less than 0.05 meant a significant difference. SPSS version 20.0 was used (SPSS Inc., Chicago, IL, USA) for analysis.

## 3. Results

### 3.1. Clinical Chemistry and Histological Examination of Renal Tissues

**Figure 1:**
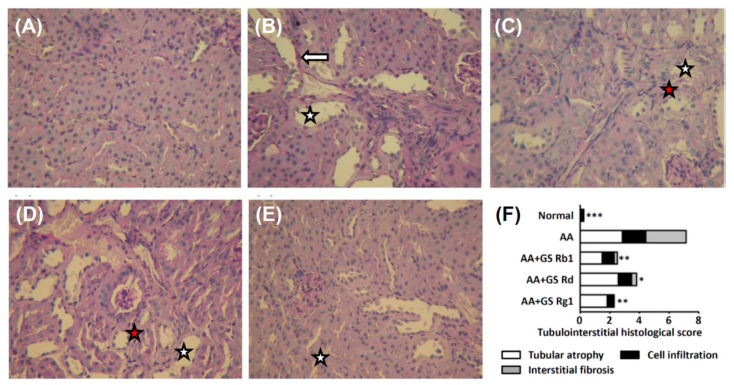
Light microscopy findings of the renal tissue of normal group (**A**), AA group (**B**), and AA plus treatment groups (5 mg/kg of ginsenoside Rb_1_, Rd, or Rg_1_) (**C**–**E**). Tubulointerstitial histological scores (**F**). The GE treated groups demonstrated the amelioration of tubulointerstitial damage, such as tubular cell atrophy (white star), cell infiltration into interstitium (red star), and interstitial fibrosis (white arrow). (PAS stain, ×200). Abbreviations: AA, aristolochic acid; GE, ginseng extract.* *p* < 0.05, ** *p* < 0.01, *** *p* < 0.001 compared with the AA group.

Compared with the normal group, the AA group had higher urinary protein, NAG, BUN, and serum creatinine levels. After co-treatment with GE 125, 250, or 500 mg/kg/d, urinary protein, NAG, and BUN levels were significantly decreased, and the serum creatinine level was significantly decreased only in AA and GE 250 mg/kg treatment groups compared with the AA group (Table S1). After co-treatment with Rg_1_, Rd, or Rb_1_, urinary protein, NAG and BUN levels were significantly decreased, and the serum creatinine level was significantly decreased in Rb_1_ and Rg_1_ groups compared with the AA group (Table 1). In the AA group, there was tubular atrophy, inflammatory cell infiltration, and interstitial fibrosis in renal tissues. After co-treatment, renal damage was alleviated by the GE treatment, especially at a dose of 250 mg/kg (Figure S2), and in Rg_1_ (Figure 1).

### 3.2. Quantification of GS Contents in GE

The quantification of GS content in GE was validated, and the results met the criterion (CV < 15%) (Table S2). The results showed that GS accounted for 2.64 to 8.10% of GE (Table S3).

### 3.3. Renal Differential Proteomics Coupled with Multivariate Analysis

**Figure 2:**
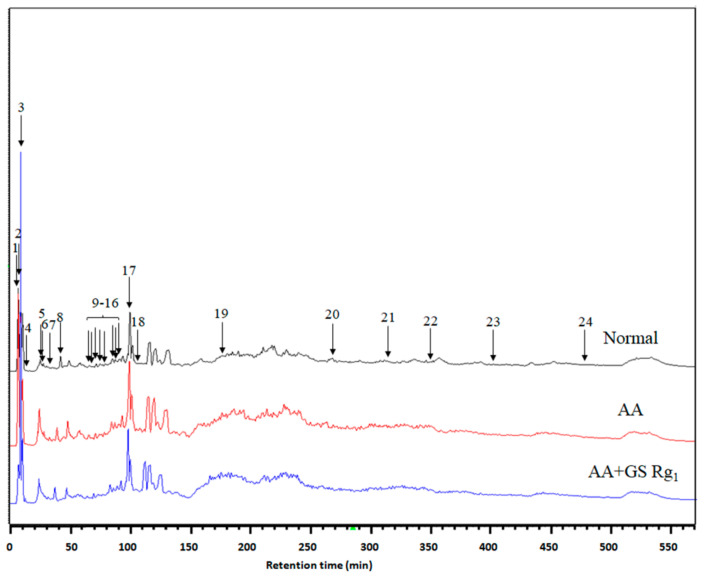
Chromatogram of FD-LC/MS/MS. Renal homogenates were analyzed using FD-LC-MS/MS. After separation, 24 peaks were found to be significantly different. PLS-DA separated protein according to classification. (a) Red: normal. Green: AA group. Blue: AA + Rg_1_ group. The score plot shows good separation. (b) Some observations were apparently discriminated in the loading plot. Abbreviations: AA, aristolochic acid; GS, ginsenosides.

**Figure 3:**
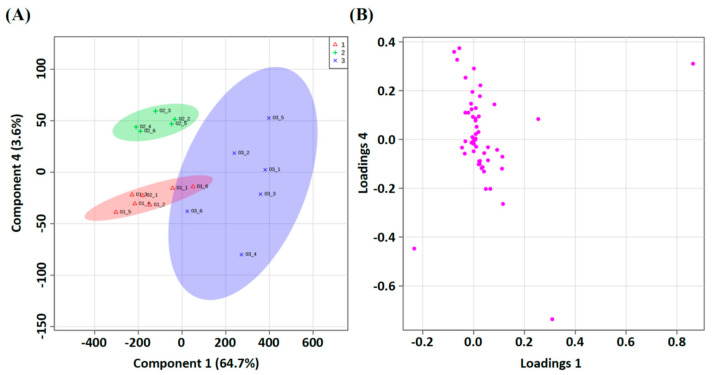
PLS-DA separated protein according to classification. (**A**) Red: Normal; Green: AA group; Blue: AA+GS Rg_1_ group. The score plot shows good separation. (**B**) Some observations were apparently discriminated in the loading plot. Abbreviations: AA, aristolochic acid; GS, ginsenosides.

We chose normal, AA, and AA with Rg_1_ (5 mg/kg/d) groups to conduct renal differential proteomics with multivariate analysis (Figure S3). The renal homogenates were analyzed using FD-LC-MS/MS. After separation, 24 peaks were significantly different between these groups (Figure 2). In the PLS-DA model, three groups were significantly separated in the score plot (Figure 3A), and several observations contributed to this model in the loading plot (Figure 3B). The LOOCV revealed that the PLS-DA model was not overfitting (Figure S4) and was suitable for the discovery of the potential biomarkers. All peaks of significance were further identified using the Mascot search engine, and the results are shown in Table 2.

### 3.4. Western Blotting Analysis

**Figure 4:**
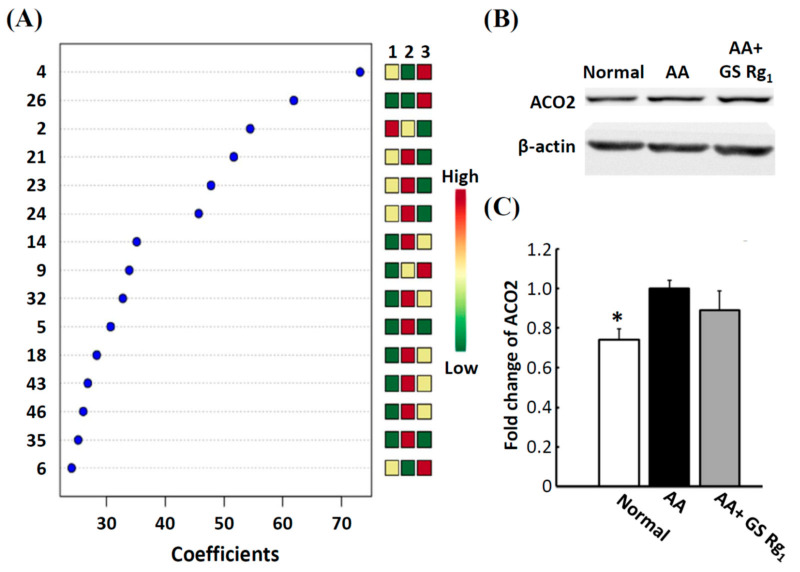
Variable importance in projection (VIP) scores of PLS-DA and quantification of ACO2 protein expression. (**A**) A VIP threshold of greater than 1 was used to identify the important biomarkers. (**B**) Protein band of mitochondrial aconitase 2 (ACO2) Western blot analysis. β-actin was used as the internal standard. (**C**) Bar graphs were constructed according to the fold change of AA group. Abbreviations: AA, aristolochic acid; GS, ginsenosides. * *p* < 0.05, compared with the AA group.

According to the coefficient score of PLS-DA, six observations were greater than 40, which were regarded as important features (Figure 4A), especially ACO2. Western blotting analysis showed that ACO2 was significantly upregulated in the AA group compared with the normal group. After Rg_1_ (5 mg/kg) co-treatment, ACO2 expression slightly decreased (Figure 4B,C). The trends of Western blotting analysis and FD-LC/MS/MS results were similar. Therefore, FD-LC/MS/MS was suitable for the discovery of the potential biomarkers.

## 4. Discussion

This study reveals that ginsenoside Rg_1_ can rescue mitochondrial disease in mice with aristolochic acid-induced nephropathy. Our results showed that administration of AA caused renal interstitial fibrosis, inflammatory cell infiltration, and proximal tubular damage. Liu et al. demonstrated that administration of AA leads to renal interstitial fibrosis accompanied by mitochondrial fragmentation in chronic AAN rats [34]. AA promotes mitochondrial DNA (mtDNA) damage; decreases mtDNA copy number, mitochondrial protein expression, and ATP content; and increases oxidative stress in cultured podocytes [35]. Mitochondrial damage leads to the release of cytochrome c into cytoplasm, which results in apoptosis [36]. Therefore, mitochondria-related proteins might have a crucial role in AAN mice.

It is well known that GE (100 or 250 mg/kg/d) attenuates renal dysfunction by alleviating advanced glycation end product-mediated with diabetic nephropathy [17]. Yokozawa et al. demonstrated that administration of GE decreases BUN levels in nephrectomized rats [37]. According to our results, administration of 250 mg/kg GE had better renoprotective effects than other doses (125 and 500 mg/kg) on chronic AAN in mice. In order to investigate the renoprotective effects of ginseng on AAN mice, we selected major components of GE, including Rg_1_, Rd, and Rb_1_, as potential candidates. All ginsenosides ameliorated AA-induced renal injury. Notably, Rg_1_ provided the best renoprotection, revealed using histopathology and clinical chemistry. Rg_1_ prevents 1-methyl-4-phenylpyridinium ion-induced apoptosis by inhibiting the production of ROS and activating the JNK pathway in SHSY5Y cells [38]. Early studies have shown that Rg_1_ (20 to 50 mg/kg) reduces the damage to the glomerular structure in spontaneously hypertensive rats by inhibiting oxidative stress, and inhibits renal interstitial fibrosis in rats with unilateral ureteral obstruction [19,39]. Rg_1_ inhibits renal interstitial fibrosis in rats with unilateral ureteral obstruction via suppressing both active tumor growth factor-β1 and phosphorylated Smad2 [40], and improves anti-glomerular basement membrane-induced nephritis in rats [41]. Therefore, we suggested that Rg_1_ had a renoprotective effect against AAN in mice.

Proteomics is a powerful tool for systematic investigation of potential biomarkers and relative mechanisms. Renal differential proteomics is a powerful tool for systematic investigation of altered proteins, used to discover a variety of potential biomarkers. FD-LC-MS/MS is one of the HPLC methods that was well-established by Masuda et al. [42] and verified by various applications [10,43]. Several biofluids have been used in differential proteomics studies, including serum, urine, and tissue homogenate. Using these, renal differential proteomics is the most suitable and direct method for the discovery of the alteration proteins in renal injury models [10,44,45]. Several groups were successful in identifying potential biomarkers in several diseases using FD-LC-MS/MS [46,47]. Using untargeted LC-MS-based metabonomics can elucidate the mechanism of AA-induced testicular toxicity [48].

In our PLS-DA analysis, six observations were most important, including peaks 2, 4, 21, 23, 24, and 26. Among these, we chose mitochondrial ACO2 (peak 21) for further Western blot analysis to validate the FD-LC/MS/MS method. According to our results, ACO2 expression was significantly increased in the AA group and decreased in the AA with Rg_1_ group. The patterns were consistent with the results of FD-LC-MS/MS. Our previous study also showed the same with Western blot analysis and FD-LC/MS/MS [10]. Therefore, FD-LC/MS/MS is suitable for the discovery of the potential biomarkers and mechanisms in the field of proteomics.

AA leads to renal glomerular defects through the activation of antioxidant enzymes, increasing oxidative stress, and causing mitochondrial dysfunction [6,35]. Mitochondrial dysfunction may be involved in AA-induced apoptosis in proximal tubular epithelial cells [8]. A recent study found that AA also can induce liver injury in rats through increasing the expression levels of apoptotic proteins caspase-9 and caspase-3, and can cause severe mitochondria damage [49]. Mitochondrial ACO2 is an enzyme that catalyzes the interconversion of citrate to isocitrate via *cis*-aconitate in the second step of the TCA cycle. Proteomic analysis of mitochondria from animal models, such as those of sepsis, diabetes, and aging, revealed that ACO2 is inactivated and nitrated [50,51,52]. One study showed that inactivation of ACO2 might prevent H_2_O_2_ and superoxide formation via the mitochondrial respiratory chain [53]. Pozdzick et al. showed that AA tubulotoxicity leads to the downregulation of antioxidative enzymes and mitochondrial damage in rats [6]. Furthermore, Li et al. also demonstrated that the antioxidative capacity is significantly decreased in AAN mice compared with the normal mice [54]. Antioxidative compounds reducing AA-generated ROS resulted in attenuation of AA-induced cytotoxicity. Mitochondrial iron overload-mediated Nrf2-HO-1/GPX4 antioxidative system inhibition would assist AA-induced ferroptosis in renal tubular epithelial cells [7].

However, there are opposite opinions showing that ACO2 is upregulated during the early stage of type 1 diabetes in the rat [55] and downregulated in 1,2-(dichlorovinyl)-l-cysteine-induced renal injury in LLC-PK1 cells [56]. The gel-based proteomics showed that the expression of ACO2 increases at first and then decreases in the unilateral ureteral obstruction model [57]. These results showed that mitochondrial aconitase might be a primary target of oxidative stress in vivo. In the AA with Rg_1_ group, the expression of ACO2 slightly decreased compared with the AA group. A study demonstrated that co-treatment with Rg_1_ (2.5 μM) recovers the inactivation of mitochondrial ACO2 activity in oxidative stress-mediated neurotoxicity in vitro. Unfortunately, the expression of ACO2 was not determined [58]. To the best of our knowledge, our study is the first one to demonstrate that AA-induced renal injury is positively correlated with the upregulation of ACO2 in kidney tissues; the detailed mechanism of ACO2 in AAN still needs to be elucidated.

AA-induced oxidative stress in mice was associated with increases in NADPH oxidase 2 (NOX2) and CYP2E1 expression, and decreases in catalase, superoxide dismutase, and glutathione synthetase expression [59]. Additionally, AA can cause mtDNA depletion, respiratory chain defects, and lower ATP content, leading to impaired respiratory complex I activity [60]. AA can also cause a depolarization of mitochondrial membrane, release of cytochrome c, and an increase of caspase-3 activity in proximal tubular epithelial cells [61]. Rapamycin protects against AA induced nephropathy by activating the mTOR autophagy axis [11].The Usmg5, known as diabetes-associated protein in insulin-sensitive tissues (DAPIT), is a part of the mitochondrial ATP synthase [62]. In human embryonic kidney 293T cells, the over-expression of Usmg5 causes mitochondrial dysfunction and activates hypoxia-inducible factor 1α and Wnt/β-catenin signaling, which results in a shift of aerobic metabolism toward a more glycolytic direction, and epithelial to mesenchymal transition [63]. In other cases, suppression of Usmg5 in HeLa cells causes loss of ATP synthase in mitochondria [64]. Therefore, we speculated that Usmg5 might play an important role in mitochondrial ATP synthesis and might correlate with renal interstitial fibrosis in AAN mice.

Cytochrome c oxidase subunit Va (COX5a) is one of the subunits of mitochondrial respiratory chain complex IV. The downregulation of COX5a is observed in type 2 diabetic mice [64]. However, gel-based proteomics showed that upregulation of COX5a occurs in renal cell carcinomas [65]. In neonatal rat kidney, COX5a is upregulated after partial unilateral ureteral obstruction [66]. Another study showed that the expression of COX5a is upregulated in breast cancer tissues compared with healthy tissues [67]. In tumor cells, Bcl-2 interacts with COX5a, which results in the alteration of mitochondrial respiration under oxidative stress [68]. Although our study did not show any renal tumors, the upregulation of COX5a might correlate with the carcinogenic activity of AA.

The present study had some limitations. First, study limitations concerned design, inherent risk of bias, and small sample sizes. Second, using GE or GS in mice with AA -induced nephropathy should be verified to ensure the rationality of the experimental grouping, which is also a limitation that should be noted for future experiment group design. Third, the experiment lacked a mitochondrial function assay.

## 5. Conclusions

In this study, we demonstrated, for the first time, that mitochondria-related proteins (Usmg5, COX5a, and ACO2) are upregulated in renal tissues in AAN mice and downregulated following Rg_1_ treatment. We speculate that the renoprotection of Rg_1_ might be ascribable to the suppression of interstitial fibrosis (Usmg5), mitochondrial respiratory chain-related protein (COX5a), and oxidative stress (ACO2). However, the possible mechanisms of these proteins remain to be elucidated in the future.

## Figures and Tables

**Table 1:** The effect of GS on clinical chemistry in chronic AAN mice.

Group	Urinary Protein (mg/Day)	NAG (μM/min/L)	BUN (mg/dL)	Creatinine (mg/dL)
Normal	1.73 ± 0.10 **	2.07 ± 0.03 **	17.69 ± 0.51 **	0.27 ± 0.05 **
AA	3.02 ± 0.09	3.85 ± 0.14	22.55 ± 1.08	0.38 ± 0.04
AA + GS Rb_1_ ^1^	2.12 ± 0.15 **	2.82 ± 0.08 *	19.50 ± 1.29 *	0.30 ± 0.01 *
AA + GS Rd ^1^	1.94 ± 0.21 *	2.97 ± 0.08 *	17.40 ± 1.52 **	0.33 ± 0.05
AA + Rg_1_ ^1^	2.00 ± 0.21 **	2.89 ± 0.04 *	20.50 ± 0.58 *	0.30 ± 0.01 *

Abbreviations: AA, aristolochic acid; AAN, AA-induced nephropathy; BUN, blood urea nitrogen; GS, ginsenosides; NAG, N-acetyl-β-D-glucosaminidase. ^1^ 5 mg/kg/d for 14 days. * *p* < 0.05, ** *p* < 0.01 compared with the AA group.

**Table 2:** Proteins identified from renal differential proteomics using FD-LC/MS/MS.

Peak No.	Protein Name	UniProtKB	Score	Compared with AA Group		Coverage (%)
				Normal	AA + Rg_1_	
19	Acyl-CoA synthetase medium-chain family member 2	Q8K0L3	50	↓	↑	1%
21	Upregulated during skeletal muscle growth 5	Q78IK2	116	↓	↓	43%
21	Aconitase 2, mitochondrial	Q99KI0	137	↓	↓	5%
24	Cytochrome c oxidase subunit Va preprotein	P12787	54	↓	↓	11%

Abbreviations: AA, aristolochic acid. ↑, Increase in the expression level; ↓, Decrease in the expression level.

## Data Availability

The data presented in this study are available on request from the corresponding author.

## Supplementary Materials

The following are available online at https://www.mdpi.com/article/10.3390/life11101018/s1. Figure S1: Box plots and kernel density plots before and after normalization. Figure S2: Light microscopy findings of renal tissue of normal group (a), AA group (b), and AA plus GE groups (125 mg/kg, 250 mg/kg, 500 mg/kg) (c, d, e). Tubulointerstitial histological scores (f). Figure S3: Quantification of Rg_1_, Rb_1_, and Rd in GE using HPLC method. Figure S4: Leave-one-out cross validation of PLS-DA model. Table S1: The effect of GE on clinical chemistry in chronic AAN mice. Table S2: The method validation for analyzing Rg_1_, Rb_1_, and Rd. Table S3: The amounts of ginsenodises in ginseng extract.
