# Supplementary material for: The Ginsenoside Rg1 Rescues Mitochondrial Disorders in Aristolochic Acid-Induced Nephropathic Mice
Source: Life (Basel). 2021 Sep 27;11(10):1018. doi: 10.3390/life11101018 (PMC8539135; doi:10.3390/life11101018)
Supplement: Supplementary file 1 [file life-11-01018-s001.zip › life-1328387-supplementary.pdf]

life

MDPI

Supplementary Materials

The Ginsenoside Rg1 Rescues Mitochondrial Disorders in Aristolochic Acid-Induced Nephropathic Mice

Chu-Kuang Chou [1], Yu-Shen Huang [2], Pei-Yu Lin [3], Kazuhiro Imai [4], Shih-Ming Chen [2] and Jen-Ai Lee [2,*]

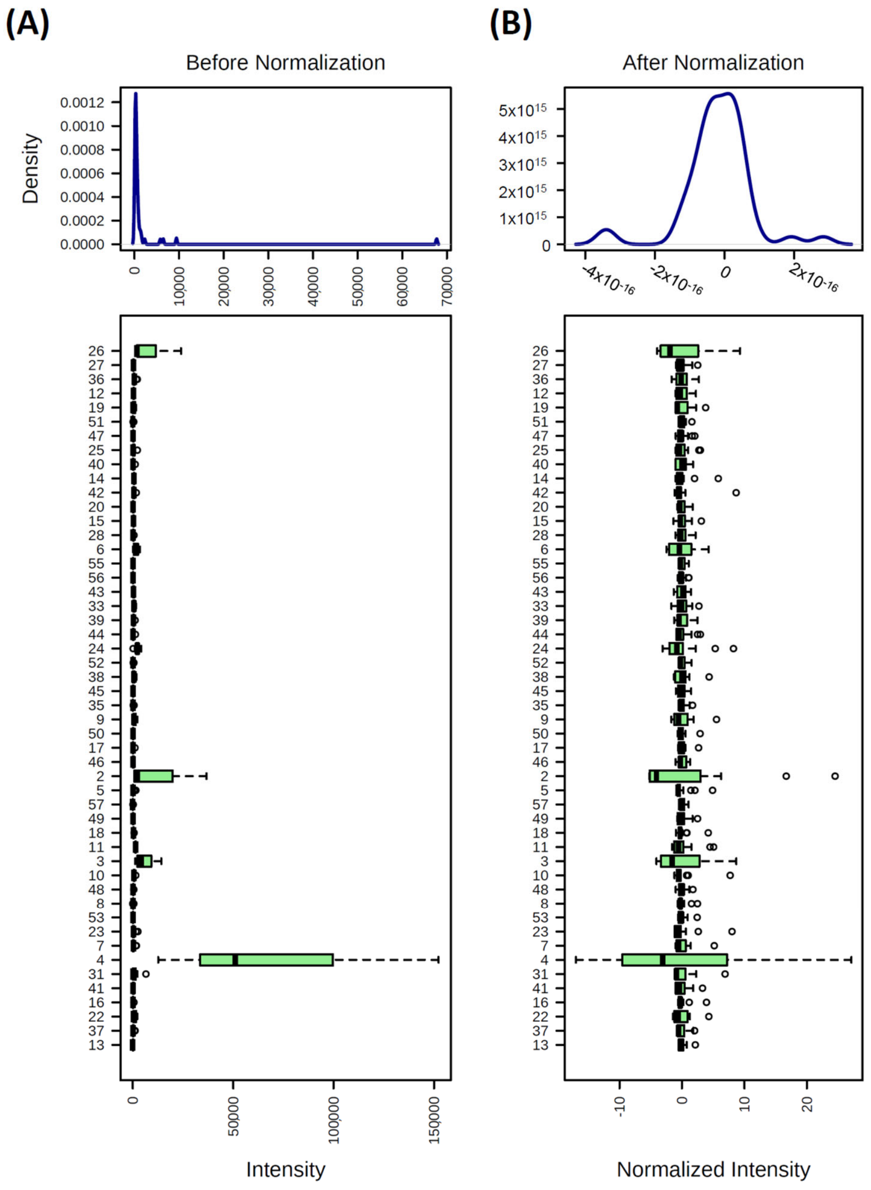

Figure S1. Box plots and kernel density plots (A) before and (B) after normalization. The boxplots show at most 50 features due to space limit. The density plots are based on all samples. Selected methods: Row- wise normalization: Normalization to sample median; Data transformation: N/A; Data scaling: Pareto Scaling.

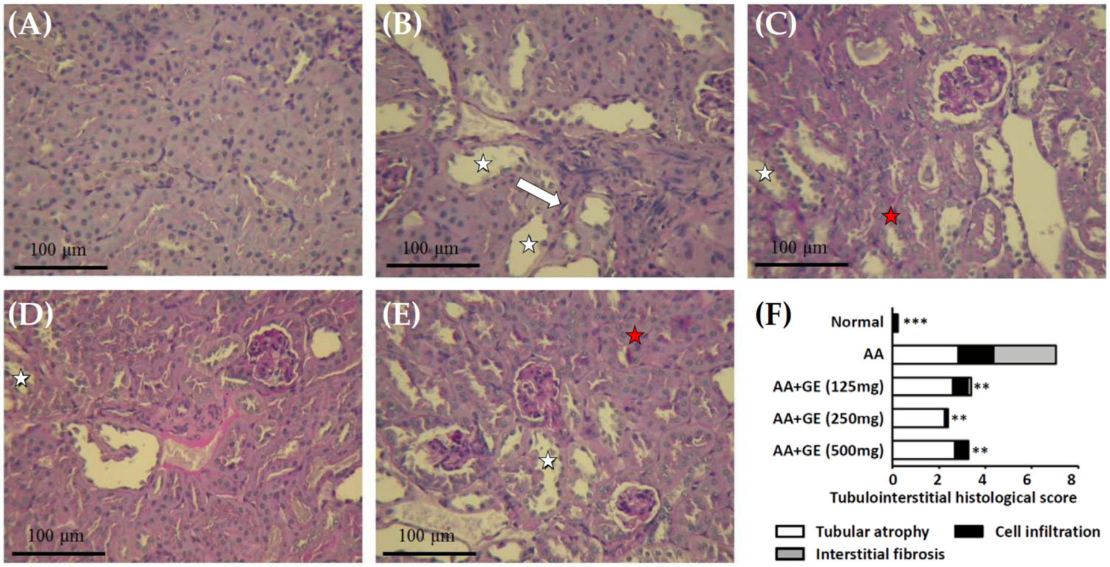

Figure S2. Light microscopy findings of renal tissue of (A) normal group , (B) AA group, (C-E) AA plus GE (125 mg/kg, 250 mg/kg, 500 mg/kg)) and (F) tubulointerstitial histological score, respectively. The GE treated groups demonstrated the amelioration of tubulointerstitial damage, such as tubular cell atrophy (white star), cell infiltration into interstitium (red star) and interstitial fibrosis (white arrow). (PAS stain, x 200). ***p*< 0.01, ****p*< 0.001 compared with the AA group.

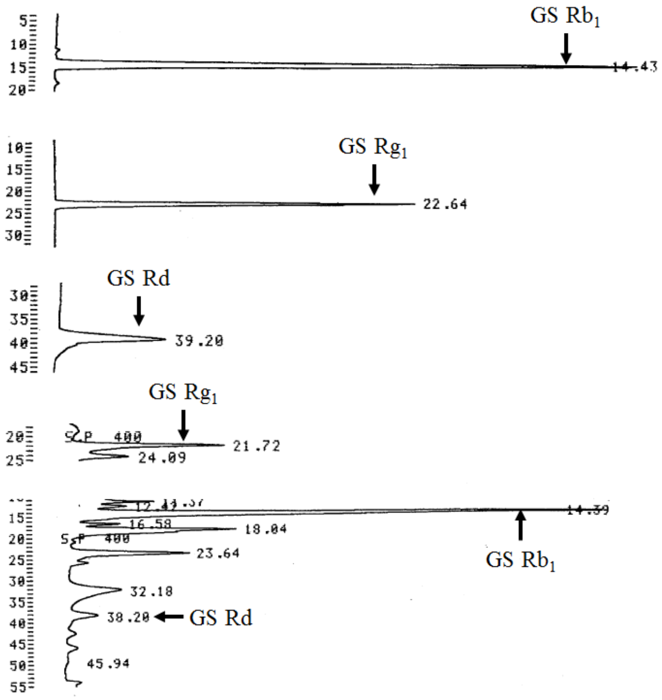

Figure S3. Quantification of GS Rg_1, Rb_1 and Rd in GE using HPLC method.

Measure	Accuracy	R2	Q2
1 comps	0.6111	0.6367	0.5506
2 comps	0.8333	0.8167	0.6597
3 comps	0.7778	0.921	0.742
4 comps	0.8889	0.9539	0.7474
5 comps	0.8889	0.9899	0.6971

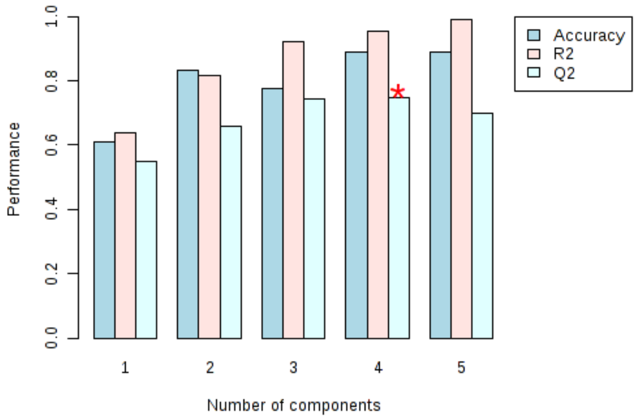

Figure S4. Leave-one-out cross validation of PLS-DA model. The star (*) indicates the best classifier.

Table S1. The effect of GE on clinical chemistry in chronic AAN mice.

	**Ginseng Extract (GE)**			
	**Urinary Protein (mg/day)**	**NAG (μM/min/L)**	**BUN (mg/dL)**	**Creatinine (mg/dL)**
Normal	1.73 ± 0.06**	2.27 ± 0.10**	18.33 ± 1.97**	0.25 ± 0.05**
AA	3.06 ± 0.08	3.39 ± 0.15	23.75 ± 2.06	0.40 ± 0.01
AA+GE (125 mg/kg)	1.89 ± 0.12*	2.66 ± 0.07*	21.25 ± 0.96	0.38 ± 0.04
AA+GE (250 mg/kg)	2.07 ± 0.03*	2.36 ± 0.08**	20.00 ± 1.22*	0.33 ± 0.04*
AA+GE (500 mg/kg)	1.83 ± 0.17*	2.46 ± 0.05**	23.33 ± 1.15	0.36 ± 0.05

**p< 0.01, ***p< 0.001 compared with the AA group.

Table S2. The method validation for analyzing GS Rg1, Rb1 and Rd.

	**GS Rg$_1$**			
	**Intra-day**		**Inter-day**	
**Standard curve (mg/mL)**	**Calculated concentration (mg/mL)**	CV (%)	Calculated concentration (mg/mL)	CV (%)
0.200	0.211 ± 0.015	6.880	0.211 ± 0.014	6.52
0.400	0.401 ± 0.017	4.270	0.396 ± 0.013	3.28
0.500	0.478 ± 0.017	3.560	0.495 ± 0.033	6.57
0.800	0.798 ± 0.028	3.470	0.790 ± 0.031	3.92
1.000	1.008 ± 0.015	1.480	1.010 ± 0.012	1.21
	**GS Rb$_1$**			
	**Intra-day**		**Inter-day**	
Standard curve (mg/mL)	Calculated concentration (mg/mL)	CV (%)	Calculated concentration (mg/mL)	CV (%)
0.200	0.195 ± 0.003	1.74	0.207 ± 0.011	5.22
0.400	0.415 ± 0.012	2.93	0.396 ± 0.012	2.99
0.500	0.488 ± 0.005	1.09	0.485 ± 0.020	4.19

Standard curve (mg/mL)	Intra-day Calculated concentration (mg/mL)	Intra-day CV (%)	Inter-day Calculated concentration (mg/mL)	Inter-day CV (%)
0.800	0.783 ± 0.035	4.49	0.807 ± 0.034	4.27
1.000	1.023 ± 0.034	3.37	0.999 ± 0.020	2.04
**GS Rd**				
	**Intra-day**		**Inter-day**	
Standard curve (mg/mL)	Calculated concentration (mg/mL)	CV (%)	Calculated concentration (mg/mL)	CV (%)
0.100	0.106 ± 0.009	8.84	0.104 ± 0.004	3.73
0.200	0.201 ± 0.006	3.04	0.201 ± 0.009	4.33
0.250	0.242 ± 0.008	3.35	0.248 ± 0.008	3.36
0.400	0.390 ± 0.007	1.90	0.386 ± 0.011	2.89
0.500	0.512 ± 0.001	0.01	0.511 ± 0.007	1.43

Table S3. The amount of ginsenodises in ginseng extract.

**Ginsenosides**	**Content of ginsenosides (mg/g)**		
	**GS Rg_1**	**GS Rb_1**	**GS Rd**
Ginsen Extract	3.51 ± 0.25	8.10 ± 0.40	2.64 ± 0.21
